# Interpretable artificial intelligence based determination of glioma IDH mutation status directly from histology slides

**DOI:** 10.1093/noajnl/vdaf140

**Published:** 2025-07-11

**Authors:** Shubham Innani, W Robert Bell, Hannah Harmsen, MacLean P Nasrallah, Bhakti Baheti, Spyridon Bakas

**Affiliations:** Department of Pathology and Laboratory Medicine, Indiana University School of Medicine, Indianapolis, Indiana, USA; Indiana University Melvin and Bren Simon Comprehensive Cancer Center, Indianapolis, Indiana, USA; Department of Pathology and Laboratory Medicine, Indiana University School of Medicine, Indianapolis, Indiana, USA; Indiana University Melvin and Bren Simon Comprehensive Cancer Center, Indianapolis, Indiana, USA; Department of Pathology and Laboratory Medicine, Indiana University School of Medicine, Indianapolis, Indiana, USA; Department of Pathology and Laboratory Medicine, Perelman School of Medicine, University of Pennsylvania, Philadelphia, Pennsylvania, USA; Department of Pathology and Laboratory Medicine, Indiana University School of Medicine, Indianapolis, Indiana, USA; Department of Computer Science, Luddy School of Informatics, Computing, and Engineering, Indiana University, Indianapolis, Indiana, USA; Department of Biostatistics and Health Data Science, Indiana University School of Medicine, Indianapolis, Indiana, USA; Department of Radiology and Imaging Sciences, Indiana University School of Medicine, Indianapolis, Indiana, USA; Department of Neurological Surgery, Indiana University School of Medicine, Indianapolis, Indiana, USA; Indiana University Melvin and Bren Simon Comprehensive Cancer Center, Indianapolis, Indiana, USA; Department of Pathology and Laboratory Medicine, Indiana University School of Medicine, Indianapolis, Indiana, USA

**Keywords:** AI, gliomas, histology, IDH, interpretability

## Abstract

**Background:**

Isocitrate dehydrogenase (IDH) mutation status is a diagnostic requirement for glioma with associated prognostic and therapeutic implications. Clinical routine visual assessment of tissue is insufficient to determine IDH status conclusively, mandating molecular workup that is unavailable everywhere.

**Methods:**

We developed an interpretable Artificial Intelligence (AI)-based approach for determining IDH status directly from H&E-stained glioma slides. Our study is based on 2442 multi-institutional whole slide images (WSIs) from 3 independent retrospective glioma collections, following their reclassification according to the WHO 2021 criteria: (1) TCGA-GBM/TCGA-LGG (*n*_WSI_ = 1534, *n*_patients_ = 799), (2) University of Pennsylvania Health System collection (UPHS, *n*_WSI_ = *n*_patients_ = 114), and (3) EBRAINS (*n*_WSI_ = *n*_patients_ = 794). Method development is based on TCGA, whereas UPHS and EBRAINS are independent hold-out datasets for model validation. Six pathology-specific foundation AI models and an ImageNet-pretrained AI model facilitate robust feature extraction. Features are aggregated into slide-level representations via an interpretable multiple-instance learning (MIL) mechanism to differentiate IDH-wildtype from IDH-mutant cases and generate attention heatmaps correlating with identifiable morphologic characteristics.

**Results:**

Our approach yields AUC_TCGA_ = 0.96 over a 10-fold cross-validation schema and generalizable performance on independent validation (AUC_UPHS_ = 0.97, AUC_EBRAINS_ = 0.95). Interpretability analysis reveals high attention regions in IDH-wildtype tumors exhibiting significant pleomorphism and microvascular proliferation, while IDH-mutant tumors show dense nodular cell concentrations, microcysts, and gemistocytic cells.

**Conclusions:**

Accurate H&E-based determination of glioma IDH mutation status can expedite conclusive diagnosis and clinical decision-making and even facilitate it in underserved regions. Finally, interpretability analysis of distilled human-identifiable features can further improve our understanding of the disease.

**Key Points**

- Isocitrate dehydrogenase (IDH) status is a glioma diagnostic requirement but mandates molecular workup.
- Our AI approach robustly determines IDH status directly from H&E-stained slides.
- Interpretability analysis identifies morphologic characteristics correlated with IDH.

**Importance of the Study**

The 2021 update to the World Health Organization classification system for central nervous system tumors integrates molecular features in diagnosing adult-type diffuse gliomas. However, not all healthcare systems have access to molecular analysis. We propose a computational approach as an alternative to predict molecular status directly from whole slide image (WSI). This study demonstrates that deep learning can accurately predict isocitrate dehydrogenase (IDH) mutation status from WSI and identify corresponding histological features through attention heatmaps for model interpretability. This work supports using digital workflows to streamline decision-making, potentially eliminating the need for molecular analysis and enabling quicker diagnoses in clinical settings.

Gliomas are the most common adult primary central nervous system (CNS) tumors,^1^ with tremendous prognostic variability. Over the last decade, our disease understanding has improved, influencing the conclusive World Health Organization (WHO) diagnostic criteria for glioma from a pure histologic assessment of morphologic features^2^ to an integrated diagnosis incorporating molecular profiling driven by distinct patient prognostication^3^ (Figure 1a). The WHO 2021 classification criteria identify IDHmut (isocitrate dehydrogenase-mutant) and IDHwt (IDH-wildtype) tumors as biologically distinct entities, rendering IDH mutation status as an important diagnostic and prognostic marker in glioma patients, indicating distinct patient management, overall survival, and clinical outcomes.^4–6^ Specifically, IDHwt glioblastoma (GBM) is differentiated from astrocytoma, IDHmut (Astros) and oligodendrogliomas, IDHmut (Oligos), especially as IDH-targeted therapies are under development.^7^ However, this distinction mandates molecular profiling and informs patient eligibility for clinical trials focused on IDHwt versus IDHmut gliomas.^8,9^

**Figure 1.**
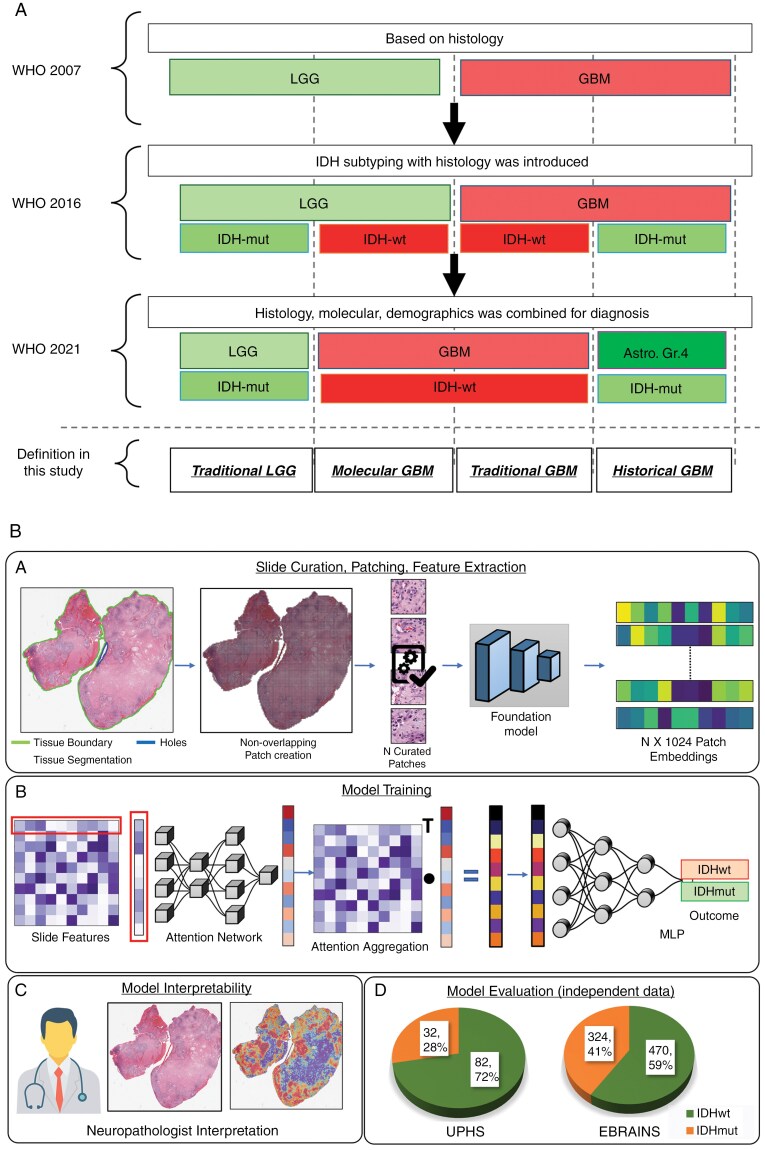
WHO 2021 reclassification along with schematic representation of the complete proposed methodological workflow. (a) Schematic representation of WHO CNS classification from 2007 to 2016 to 2021. (b) (A)WSI pre-processing from segmentation to patching to feature extraction with SSL-based pre-trained model (B) Model training with attention-based multiple instance learning for slide-level classification into IDHwt or IDHmut (C) Attention heatmaps interpretation by neuropathologist.

Histomorphologic correlations with molecular alterations are well established in diffuse gliomas.^10^ Certain histopathological features are strongly associated with IDHmut status, such as gemistocytic and oligodendroglial-like cytomorphology.^11,12^ In contrast, higher-grade characteristics like palisading necrosis and microvascular proliferation are enriched in IDHwt tumors.^13^ However, these associations are not absolute, and a subset of IDHwt GBM do not have Grade 4 histologic features and are microscopically indistinguishable from lower-grade astrocytoma, IDHmut. This study defines these tumors as “molecular” GBM (mGBM)^14,15^ if they exhibited molecular abnormalities of: TERT promoter mutation, EGFR amplification, or chromosomal +7/−10 copy changes.^3^

While some studies highlight the potential of neuroimaging in predicting IDH mutation status,^16^ these approaches have yet to demonstrate diagnostic performance comparable to histopathology or molecular profiling^17^ due to numerous factors,^18,19^ including variability in imaging protocols, lack of standardization, and importantly gliomas’ microenvironmental heterogeneity. Consequently, tissue sampling through surgical resection remains a pivotal aspect of current treatment strategies.^20^ Following surgical sampling, the gold standard for detecting IDH mutations involves immunohistochemical staining, typically employing the IDH1-R132H antibody^21^ and/or sequencing.^22^ However, these methods can be expensive and time-demanding, often necessitating outsourcing or, when unavailable, leading to the “IDH-non-otherwise-specified” (IDH-NOS) label, which affects clinical/therapeutic decision-making.^23,24^

Recent advances in artificial intelligence (AI) for computational neuro-oncology have revolutionized the analysis of whole slide images (WSIs) for clinical endpoints, with the potential to automate clinical diagnosis, predict patient prognosis and response to therapy, and discover new morphological biomarkers.^19^ The analysis of WSIs remains indispensable in precision oncology, providing vital diagnostic information for various tumor types, especially when interpreted by expert neuropathologists.^25^ Robust deep learning (DL) algorithms that harness the wealth of existing and prospective WSI have the potential to (1) assist pathologists in delivering precise subspecialty diagnoses, (2) serve as reliable, fatigue-free quality checks to minimize human error, and (3) unveil morphological patterns associated with biomarkers, paving the way for enhanced patient care even in community settings.

The rapid advancement in computer processing power and developments in AI models have significantly enhanced our ability to extract valuable insights from WSIs.^26,27^ This progress has notably impacted glioma research by exploring downstream tasks like prognostic stratification,^28^ biomarker identification,^29,30^ and histology subtyping.^31^ Further advances in AI for computational pathology employ multiple-instance learning (MIL) techniques to enable training on WSIs in a weakly supervised manner,^32,33^ overcoming the limitations caused by the patch selection process. In these approaches, WSIs are divided into smaller patches and represented as feature vectors using feature extractors, recently utilizing foundation models (FMs).^34^ MIL models are effectively handling slide-level labels for WSIs without detailed annotations.^32^ The successful application of weakly labeled WSIs highlights the need for strong FMs to boost the performance of MIL-based models.^33,35,36^ Recent FM methods rely on self-supervised learning (SSL) models, trained on millions of patches from thousands of WSIs from large-scale datasets using various algorithms, including Vision Transformer-based clustering methods^37^ and contrastive learning.^38–41^ These models have become popular in computational pathology due to their effectiveness with large, non-annotated datasets and their ability to outperform traditional ImageNet^42^ pre-trained models in downstream tasks like cancer subtyping, diagnostics, prognostics, and predictive modeling.^25,32,43^

In this paper, recognizing the prognostic significance of IDH mutation status in glioma, we present an AI approach for its robust prediction from H&E-stained WSIs alone. We develop and evaluate our algorithm on retrospective glioma collections with a well-defined patient population from TCGA,^44^ EBRAINS,^45^ and UPHS reclassified by board-certified neuropathologists as per WHO 2021 criteria. During model development, patient subcohorts are created based on their histologic characteristics/grade for ablation studies, to avoid algorithmic bias based on grades and ensure sub-visual cues of distinct molecular status are identified even across cases of similar morphology. The model developed based on weakly supervised MIL methodology can be divided into 2 steps: (1) feature extractor with six pathology-specific FMs and (2) feature aggregation into slide-level representation with 5 MIL strategies. We further conduct interpretability analysis linking high-attention regions to IDH-associated histopathology validated by neuropathologists. This approach unveils essential glioma morpho-molecular correlations, delving into their clinical significance and enriching our understanding of IDH mutation identification for improved prognostic assessments.

## Materials and Methods

### Data

The evaluation and interpretation of our proposed work are based on 2442 multi-institutional WSIs from publicly and privately available data collections. The model development is carried out on publicly available multisite retrospective patient populations from TCGA-GBM and TCGA-LGG collections, accessed through The Cancer Imaging Archive (TCIA).^44^ As frozen sections include hydration artifacts due to freezing, this study’s analysis focuses only on formalin-fixed paraffin-embedded WSIs. WSIs from TCGA have either 40× (mpp: 0.2456–0.2533) or 20× (mpp: 0.4993–0.504) maximum apparent magnification. To ensure uniformity and consistency, 20× magnification was considered the standard reference level for comparison in subsequent analyses. To assess model performance, we employed a 10-fold cross-validation strategy stratified on the patient level to account for subjects with multiple WSIs to avoid potential patient-wise leakage. In addition to TCGA, we also use 2 independent out-of-sample hold-out validation cohorts from the University of Pennsylvania Health System (UPHS, *n*_WSI_ = *n*_patients_ = 114, IDHwt/IDHmut = 82/32, mpp: 0.2206) and the EBRAINS dataset (*n*_WSI_ = *n*_patients_ = 794, IDHwt/IDHmut = 470/324, mpp: 0.2278).^45^ Details for the datasets are tabulated in Table 1.

**Table 1:** Classwise Distribution of 3 Distinct Subcohorts Controlling Grade and Histology

Data used for	Subcohort	Total WSIs (patients)	IDHwt WSIs (patients)	IDHmut WSIs (patients)
Model training	**#1** GBM (Gr.4), Astros(Gr.2,3,4), Oligos (Gr.2,3)	1534 (799)	756 (379)	778 (420)
	**#2** GBM (Gr.4), Astros (Gr.2,3,4), Oligos (Gr.3)	982 (517)	698 (340)	284 (177)
	**#3** GBM (Gr.4), Astros (Gr.3,4), Oligos (Gr.3)	820 (405)	687 (321)	143 (84)
Independent hold-out validation	UPHS	114 (114)	82 (82)	32 (32)
	EBRAINS	794 (794)	470 (470)	324 (324)

During model training, our approach includes comprehensive data partitioning motivated by the distinct histologic characteristics of tumors across different grades and histologic subtypes. However, IDHmut cases are predominantly found in lower-grade gliomas, while IDHwt cases are associated with G4 GBM. Hence, our model might perform well in distinguishing IDHmut from IDHwt cases by inadvertently focusing on grade or histology. To address this concern and ensure our AI model identifies sub-visual cues of distinct molecular status even across cases of similar morphology, we carefully considered the grade and histology of gliomas while creating three distinct data subcohorts (refer Table 1). The first subcohort consists of GBM (Gr.4), Astros (Gr.2, 3, and 4), and Oligos (Gr.2 and 3), while the second subcohort consists of GBM (Gr.4), Astros (Gr.2, 3, and 4), and Oligos (Gr.3), and the third subcohort consists of GBM (Gr.4), Astros (Gr.3 and 4), and Oligos (Gr.3).

### Patching and Feature Extraction

The initial step in our methodology involves tissue segmentation (Figure 1b) to distinguish apparent tissue from the slide background. To facilitate this segmentation, we convert a downsampled (by a factor of 16) version of the WSI from RGB to the Hue, Saturation, and Value (HSV) color space. Subsequently, a binary mask is created by applying an initial median blur, followed by median filter-based blurring and morphological closing to reveal a tissue-occupied region mask. This mask is the foundation for further patch extraction of size 256 × 256 at a 20× magnification level. Additionally, we employ a comprehensive patch-level curation approach,^27,28^ allowing us to exclude patches containing artifacts, such as glass reflections, tissue folding/tearing, and pen markings, among other artifacts. All available patches from each slide were utilized to ensure comprehensive feature representation. This approach allows us to retain as few as 85 patches and up to 37 919 patches in the largest WSI, with an average of 10 104 patches per WSI.

From each patch extracted from the WSI, features are computed using the FM, resulting in a 1-dimensional vector per patch. This yields a matrix of size (*N* × *M*), where *N* is the number of patches, and *M* is the feature embedding dimension, which varies depending on the model used. Most recent MIL-based approaches for computational pathology rely on compressed WSI feature embeddings. Hence, identifying a robust feature extractor is crucial for the model’s precision.^35^ To begin with experimentation, features are extracted using (1) an Imagenet-trained ResNet50 and (2) a series of FMs. The latest FMs leverage Vision Transformers (ViT),^46^ significantly advancing feature extraction capabilities. These FMs are trained on millions of patches from thousands of WSIs from large scale datasets.^47,48^ FMs in our experimentation include CTransPath,^39^ which builds upon the ViT named MoCov3,^49,50^ RetCCL,^37^ a contrastive learning framework based on MoCov3 and ResNet-50,^51^ the Lunit model based on DINO,^38,52^ SimCLR,^41^ a contrastive learning-based ResNet18 model, and HIPT4K,^40^ a hierarchical ViT. We also explored the latest FM in pathology, UNI, trained on 100 K WSIs.^34^ The selection of 7 models was motivated by the goal of conducting a comprehensive evaluation of diverse open-source FM for weakly supervised learning. The choice of FMs represents top-performing models identified in the literature at the time of experimentation. Detailed information about these models is provided in Table 2. The FMs used in our study are publicly available and were downloaded from their original repositories using the latest available versions at the time of study. All models were implemented in Python using PyTorch, with ImageNet pretraining weights obtained through the standard PyTorch model zoo, which is a collection of pretrained weights of neural network models. We adhered to each model’s recommended preprocessing pipeline when generating features to ensure consistency and reproducibility. Additionally, to ensure reproducibility of the models, the code is made available through GitHub at https://github.com/IUCompPath/IDHclassifier.

**Table 2:** Overview of SSL Feature Extractors Evaluated in This Study, Their Architecture, SSL Method, Pretraining Dataset, Tiles in Million Indicated as M, Slides in Thousands Indicated as K, Total Number or Organs, Embedding Size

Name	Architecture	SSL method	Tiles(M)	Slides(K)	Organs	dataset	Embedding dim
ImageNet	ResNet50	-	-	-	-	ImageNet	1024
SimCLR	ResNet18	Contrastive learning	21	25	25	TCGA, CPTAC	512
HIPT4K	ViT-S	DINO	104	11	33	TCGA	192
LunitViT	ViT-S	DINO	33	37	25	TCGA, TULIP	384
CTransPath	Swin Transformer	MoCov3 based semantic contrastive learning	16	32	25	TCGA, PAIP	768
UNI	ViT-S	DINO	100	100	32	Mass100K	1024
RetCCL	ResNet-50	MoCov3 based clustering-guided contrastive learning	16	35	32	TCGA, PAIP	2048

As baselines, we additionally compare against the respective ImageNet-pretrained backbones.

### Weakly Supervised Learning

After feature extraction, we utilize a MIL framework enhanced with an attention mechanism (AttMIL)^32,53^ to consolidate patch-level features into comprehensive slide-level representations, which are pivotal for our final predictions. In this framework, the attention network assigns an “attention” score to each patch, indicating its relative importance in contributing to the overall slide-level prediction. Additionally, a binary clustering layer with 512 hidden neurons, combined with an support vector machine (SVM) loss function, follows the initial fully connected layer to refine specific features.^32^ This layer leverages features from patches with high attention scores to construct a rich slide-level feature space that effectively distinguishes between positive and negative instances for the 2 classes. Subsequently, a fully connected layer aggregates predictions from patches based on their attention scores, effectively filtering out contributions from less significant patches. This aggregation process results in the final classification of each WSI into 1 of 2 categories: IDHwt or IDHmut. The details of the attention mechanism are available in Supplementary Material (Section 1). The model was trained using the Adam optimizer with a learning rate of 0.0001, a dropout rate of 0.25 to prevent overfitting for a maximum of 200 epochs, and model checkpoints were saved based on the best validation performance. Early stopping was applied to halt training once the validation loss no longer improved after 20 consecutive epochs. Weighted cross-entropy loss is implemented to mitigate the class imbalance problem. Performance was evaluated using 2 primary metrics: the area under the curve (AUC) and the Youden index. Additionally, ablation experiments were conducted to assess the impact of the attention mechanism by comparing it against max and mean pooling strategies. The weakly supervised models tested in the ablation study included MaxMIL, MeanMIL, DSMIL, TransMIL, and AttMIL.

### Interpretability: Plausible Features Can Be Linked to Predictions

To further enable the interpretability of the predictions, we generate heatmaps that visually represent attention scores, highlighting the regions of interest (ROIs) in each WSI. The attention heatmaps for WSIs were generated using the attention scores derived from individual patches processed by the trained model. To create the attention heatmap for the entire slide, the attention scores from each patch were mapped back onto their corresponding locations in the original WSI. Once the attention scores were spatially reconstructed and visualized as a heatmap, these visualizations helped identify the morphological characteristics influencing algorithmic decisions. To comprehensively assess our model’s performance, 2 expert neuropathologists (W.R.B. and H.H.) conducted a qualitative evaluation of the generated heatmaps. This evaluation would provide valuable insights into the interpretability of the model’s predictions and highlight areas of clinical significance.

## Results

**Figure 2.**
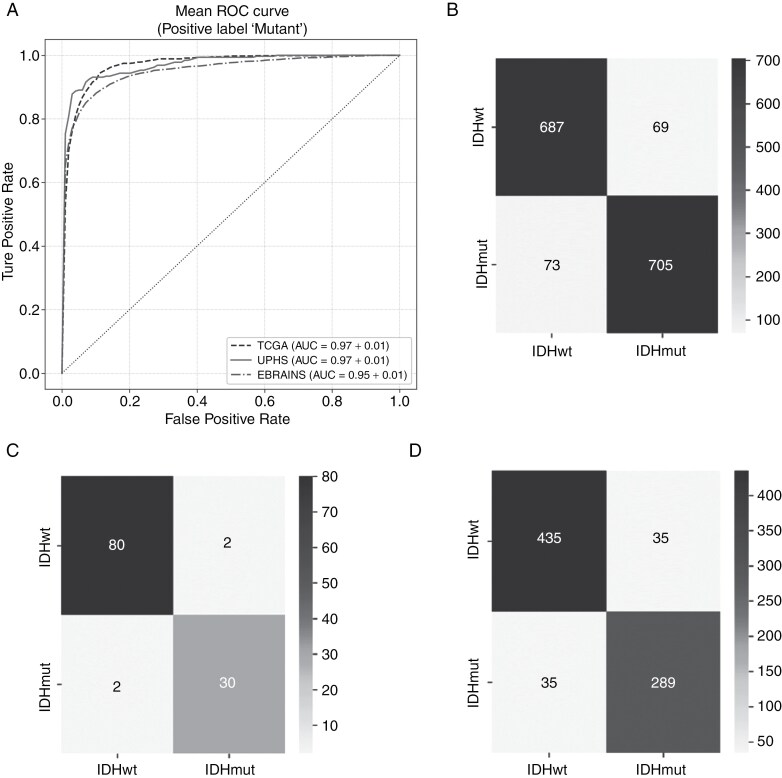
(a) AUC-ROC curve for 10 folds on TCGA cohort, UPHS cohort and EBRAINS cohort. The X-axis is the true positive rate represents sensitivity, and the Y-axis is the false positive rate represents 1 minus the specificity. (b) Confusion matrix for TCGA cohort (c) Confusion Matrix for UPHS cohort (d) Confusion matrix for EBRAINS cohort.

The comparative evaluation of our proposed approach for IDH mutation prediction is presented in Table 3a in terms of mean AUC for different feature extractors across different dataset cohorts (as in the second section) and in terms of the Youden index in Table 3b. The results presented in Table 3 for Subcohort #1, Subcohort #2, and Subcohort #3 correspond to the outcomes of the 10-fold cross-validation experiments conducted individually on each subcohort, as described in the Data section. Among the 3 subcohorts, Subcohort #1 yielded the best performance during training. The model trained on this subcohort using a 10-fold cross-validation strategy was subsequently evaluated on 2 independent hold-out validation sets, UPHS and EBRAINS. We observe an approximately 10% increase in AUC and around 25% increase in the Youden index on the hold-out UPHS and EBRAINS datasets (Table 3a), demonstrating the generalizability of SSL-based features compared to ImageNet features. Figure 2a visually represents the AUC curve for the complete TCGA data and for the independent hold-out UPHS and EBRAINS cohorts. This illustrates the consistent performance across diverse dataset cohorts, demonstrating the robustness and effectiveness of SSL-based feature extraction. Following the evaluation of the FMs, UNI emerged as the top-performing FM. We then fixed the feature extractor to UNI and evaluated the performance of different MIL strategies across three TCGA glioma cohorts. The results of this comparison are presented in the supplementary Material (Section 2).

**Table 3:** Performance Across Feature Extractors With Subcohort #1, Subcohort #2, and Subcohort #3 Correspond to the Outcomes of the 10-Fold Cross-Validation Experiments Conducted Individually on Each Subcohort Across Subcohorts

(a) Average AUC values with different feature extractors.					
	10-Fold cross-validation			Independent hold-out validation	
Model	Subcohort #1	Subcohort #2	Subcohort #3	UPHS	EBRAINS
Imagenet	0.9241 ± 0.0349	0.9054 ± 0.0417	0.8729 ± 0.0779	0.8811 ± 0.0058	0.8534 ± 0.0187
SimCLR	0.9295 ± 0.0179	0.9012 ± 0.0346	0.8801 ± 0.0560	0.8739 ± 0.0298	0.8492 ± 0.0303
HIPT 4K	0.9334 ± 0.0212	0.9281 ± 0.0461	0.9283 ± 0.0598	0.9023 ± 0.0183	0.8650 ± 0.0161
RetCCL	0.9253 ± 0.0282	0.9053 ± 0.0528	0.8779 ± 0.0872	0.9244 ± 0.0290	0.8987 ± 0.0117
CTransPath	0.9496 ± 0.0187	0.9329 ± 0.0405	0.9409 ± 0.0389	0.9553 ± 0.0185	0.9361 ± 0.0116
LunitViT	0.9552 ± 0.0118	0.9484 ± 0.0276	0.9353 ± 0.0527	**0.9760 ± 0.0088**	0.9502 ± 0.0129
UNI	**0.9716 ± 0.0113**	**0.9616 ± 0.0342**	**0.9689 ± 0.0211**	0.9737 ± 0.0074	**0.9534 ± 0.0102**

(b) Average Youden index values with different feature extractors.					
	10-Fold cross-validation			Independent hold-out validation	
Model	Subcohort #1	Subcohort #2	Subcohort #3	UPHS	EBRAINS
Imagenet	0.6927 ± 0.1017	0.6465 ± 0.0993	0.5735 ± 0.1796	0.5916 ± 0.0892	0.5576 ± 0.0557
SimCLR	0.6998 ± 0.0562	0.5924 ± 0.0656	0.5033 ± 0.1995	0.4943 ± 0.1091	0.4772 ± 0.1165
HIPT 4K	0.7161 ± 0.0446	0.6782 ± 0.0992	0.6544 ± 0.1903	0.6229 ± 0.0253	0.6022 ± 0.0015
RetCCL	0.6994 ± 0.1041	0.6041 ± 0.1075	0.5123 ± 0.2577	0.6637 ± 0.0771	0.6597 ± 0.0274
CTransPath	0.7769 ± 0.0597	0.6946 ± 0.1314	0.6530 ± 0.1888	0.7023 ± 0.0608	0.7095 ± 0.0322
LunitViT	0.7790 ± 0.0674	0.7275 ± 0.0902	0.6531 ± 0.1806	**0.8329 ± 0.0422**	0.7130 ± 0.0347
UNI	**0.8189 ± 0.0300**	**0.8093 ± 0.0735**	**0.7712 ± 0.1669**	0.8149 ± 0.0754	**0.7175 ± 0.0622**

The model trained on Subcohort #1, using the 10-fold cross-validation strategy, was then independently evaluated on 2 separate hold-out validation sets, UPHS and EBRAINS, based on the results from this cross-validation. Values depict mean ± standard deviation.

**Figure 3.**
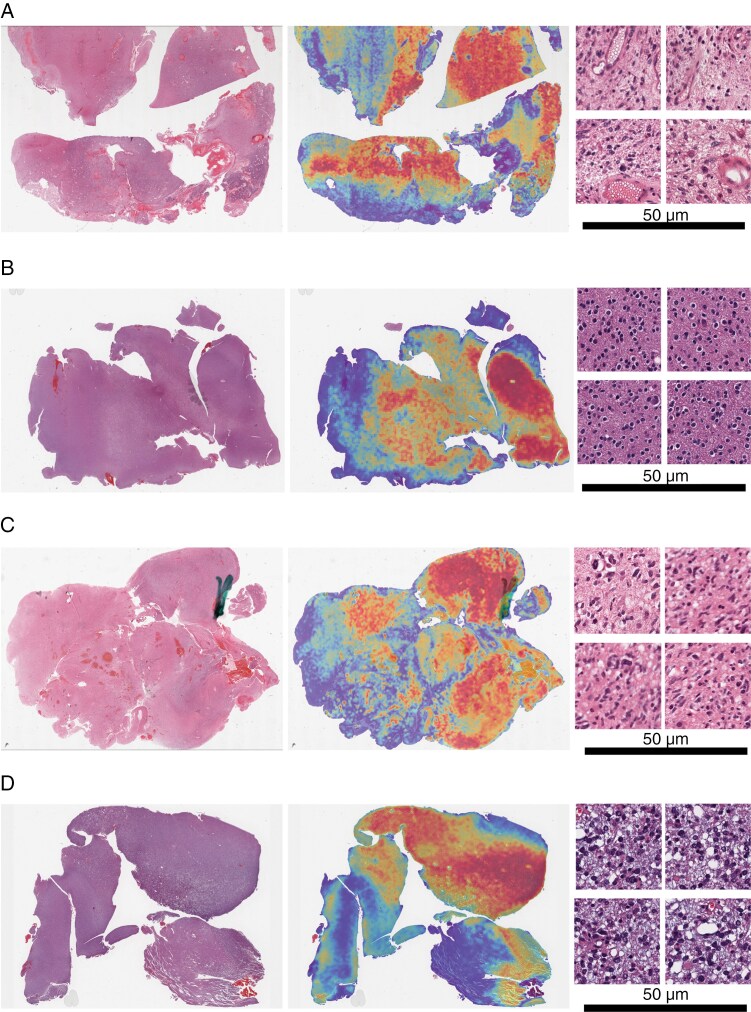
Whole slide image (WSI) with the generated attention heatmaps for interpretability, where high attention (red) corresponds to regions contributing to the model’s prediction. Each subfigure shows a WSI, its corresponding heatmap, and four sample patches with high attention for different scenarios. The right-hand panels are shown at 20× magnification, and a scale bar of 50 μm is shown.

In addition to quantitative evaluation in terms of classification performance, attention heatmaps were qualitatively assessed. Two expert neuropathologists (W.R.B. and H.H.) reviewed 24 attention heatmaps from the TCGA test dataset to identify the relevant morphologic features in IDHwt and IDHmut WSIs. To quantitatively evaluate the inter-rater reliability between the 2 board-certified neuropathologists (W.R.B. and H.H.), we computed Cohen’s Kappa coefficient,^54^ a statistical measure commonly used to evaluate the level of agreement between 2 independent raters. Each rater provided annotations for regions of high attention, describing histopathologic features of tumor tissue. These annotations included descriptive terms (refer Supplementary Material (Section 5) for all annotations) related to tumor cell morphology (eg, pleomorphism, hyperchromatic nuclei), architectural patterns (eg, microcystic change, palisading necrosis), and background characteristics (eg, fibrillary stroma, reactive epithelial cells). Each annotation was transformed into a binary representation, indicating the presence or absence of specific terms. Pairwise comparisons were then performed for corresponding annotations, and Cohen’s Kappa was computed for each pair to quantify agreement beyond chance. The average Cohen’s Kappa across all annotation pairs was 0.6096, indicating a substantial level of agreement between the 2 raters. Figure 3a–d provides illustrative examples of WSI alongside their corresponding attention heatmaps and sample patches with high attention, representing regions of high importance in the algorithm’s final decision-making process. These heatmaps serve as essential tools for visualizing and comprehending the morphological patterns and their relative importance within each region of the WSI.

## Discussion

The latest guidelines of WHO classification criteria for glioma^3^ require reporting IDH mutation status for conclusive diagnosis, with beneficial prognostication of IDHmut patients compared with IDHwt ones. In this study, we introduced a robust interpretable AI approach to determine the IDH mutation status (IDHmut vs. IDHwt) in adult diffuse glioma directly from their H&E-stained WSIs alone, following a systematic evaluation of 6 FMs along with attention-MIL-based aggregation of these features into slide-level representations. Quantitative results with a minimum AUC > 0.95 across independent multi-institutional hold-out datasets indicate our proposed approach’s robustness and potential impact (Table 3). Although clinical routine visual assessment of H&E-stained slides is inconclusive to determine IDH status and molecular profiling is required, our AI approach identifies quantitative cues enabling the successful classification of IDHwt and IDHmut cases solely from H&E-stained slides.

Although prior studies in the literature (refer Supplementary Material [Section 6]) have showcased the potential of AI models in predicting IDH mutation status from H&E WSIs, they have several limitations.^13,55–58^ For instance, Momeni et al. reported an AUC of 0.94 utilizing deep recurrent attention models on the TCGA-GBM and TCGA-LGG datasets, but they sparsely assessed WSIs based on random patch selection.^55^ Liechty et al. achieved an AUC of 0.921 by ensemble-averaging 4 AI models trained on patches from four magnification levels, albeit sparsely assessed WSIs based on random patch selection.^13^ Liu et al. employed generative adversarial networks for data augmentation, attaining an AUC of 0.927, but their study was constrained by a small dataset of 266 cases used for patch-level training.^57^ Jiang et al. specifically trained their model for Grade 2 diffuse gliomas with a cohort of 296 patients,^56^ achieving an AUC of 0.739. However, it lacked validation across other glioma grades since it was conducted before the WHO 2021 criteria. Hewitt et al.^58^ employed a weakly supervised approach trained on an internal cohort and reported an AUC of 0.95, but their model was not validated in challenging scenarios such as molecular GBM or historical GBM cases. Moreover, most of these models were trained at the patch level using a single-fold strategy, which may limit generalizability. A critical limitation across all these approaches is the lack of interpretability in the prediction outcomes, which hinders the ability to gain deeper insight into disease-relevant morphological patterns.

Our study addresses these limitations by developing and robustly evaluating an AI algorithm designed to overcome the key challenges identified in the literature. The ablation 10-fold strategy evaluation experiments of FMs highlight UNI as the top-performing model with a remarkable 10% increase in AUC on both the independent hold-out datasets (UPHS and EBRAINS) compared with the ImageNet-based baseline model. The ability of the UNI FM to achieve consistently high AUC values is likely due to its extensive training on 100 million patches derived from 100 thousand slides, combined with the advanced architecture of ViTs and a large embedding size of 1024 dimensions. Similarly, LunitViT also exhibited robust performance, attaining an AUC of approximately 0.97 on the UPHS dataset, the highest among all FMs. Despite its relatively smaller embedding size of 384 and fewer training patches, Lunit’s results suggest that high embedding dimensions are not the sole determinant of model efficacy. UNI and LunitViT outperform the other models, and one potential factor behind their superior performance is that both are based on DINOv2, a state-of-the-art SSL framework. In contrast, other models are based on earlier training frameworks, which may limit their ability to capture complex features effectively. Notably, LunitViT’s strong performance, despite using fewer WSIs in pretraining, further highlights the potential of the DINOv2 framework, suggesting that it can learn robust features more efficiently, even with smaller datasets. These findings highlight the importance of both advanced architecture and diverse, domain-relevant training data in achieving state-of-the-art performance in glioma classification.

We further explored various aggregation strategies, including MeanMIL, MaxMIL, TransMIL, and DSMIL. Our finding suggests that FMs substantially enhance MIL performance, indicating that strong feature embeddings contribute more significantly to model performance than the choice of aggregation strategy. Additionally, incorporating attention improves performance by approximately 2% compared to traditional pooling-based approaches such as MaxMIL and MeanMIL. Notably, AttMIL not only boosts predictive accuracy but also facilitates interpretability by generating attention maps that highlight the most relevant patches in the WSI. These attention scores are visualized as heatmaps, providing insights into the model’s decision-making process, an advantage not possible with basic pooling methods. The detailed results are provided in the Supplementary Material (Section 2).

The quantitative evaluation of data partitioned in subcohorts of varying grading (Gr.2–Gr.4) and histologic subtypes, including GBM, Astros, and Oligos (Materials and Methods section), highlights the ability of our proposed approach to detect the IDH mutation status from WSI alone, instead of learning traditional morphological features that describe different grades. The best performing FM (UNI) yields AUCs of 0.9716, 0.9737, and 0.9534 on Subcohort 1, the independent hold-out validation data of UPHS, and that of EBRAINS, respectively, and is subsequently used for further analysis. While comparing the performance across different subcohorts (Table 3), we note only a slight decline in performance when segregating the dataset into Subcohort 2 and Subcohort 3. This indicates the model’s robustness that irrespective of grade and histology, our model excels in learning IDH status with high accuracy based only on histology features. However, the performance decline could be attributed to the reduced number of cases and class imbalance of Subcohorts 2 and 3 compared to Subcohort 1, as indicated in Table 1.

Our subgroup analysis for grade in Subcohort 1 demonstrates that the model effectively captures IDHmut and IDHwt properties irrespective of tumor grade, reinforcing its robustness in IDH classification. The model achieved high performance across different subgroups, correctly classifying 94% of Grade 2 IDHmut tumors (Traditional LGG), 92% of Grade 3 IDHmut tumors, 62% of Grade 4 IDHmut tumors (Historical GBM), 94% of Grade 4 IDHwt tumors (Traditional GBM), and 56% of Grade 2,3 (now Grade 4 as per WHO 2021) IDHwt tumors (Molecular GBM). These results indicate that while the model does not explicitly learn tumor grades, it effectively distinguishes between IDHmut and IDHwt cases across different histologic presentations. The variation in performance across subgroups, particularly in molecular GBM and historical GBM, suggests that certain subtypes may present more histological complexity, requiring further refinement of computational approaches. The tabular version of the results is available in Supplementary Material (Section 6).

The changes in the diagnostic classification criteria of CNS tumors from pure histologic features in 2007 to an integrated histologic-molecular approach in 2021 (Figure 1a) identify some tumors with low-grade histologic features as GBM due to their molecular characterization of IDHwt (ie, molecular GBM) and, on the other hand, tumors with high-grade histology as astrocytoma, IDHmut, Gr.4, due to their molecular characterization of IDHmut, which were previously considered to be GBM (ie, historical GBM). The diagnosis of such cases based only on microscopic evaluation remains inconclusive, as identification of IDH mutation status further requires molecular assay or next-generation sequencing. Since our approach is purely based on H&E-WSI, we anticipated that the proposed algorithm would identify (1) all molecular GBM cases as IDHmut and of lower grade, and (2) all historical GBM cases as IDHwt, hence failing to correctly classify any of these two categories that require molecular profiling. Interestingly, our results reveal that the AI model correctly identified 62% of historical GBM cases as IDHmut, as compared to traditional LGG cases, where it correctly identified 93% as IDHmut. Similarly, the model correctly classified 56% of molecular GBM cases as IDHwt, compared to a 94% accuracy for traditional GBM cases. These results suggest the presence of subvisual cues in H&E-stained slides that may provide diagnostic information beyond what is discernible to the human eye. While this correct identification of molecular GBM and historical GBM was a positive finding, the observed lower performance for these subgroups when compared to the classification of traditional GBM and traditional LGG is likely due to the shared histologic features between molecular GBM and traditional LGG, as well as between historical GBM and traditional GBM.

To assess the statistical significance of performance differences among FMs, we conducted a pairwise *P*-value analysis (refer Supplementary Material [Section 4]). UNI exhibited significant differences compared to LunitViT (*P* = .0270) and CTransPath (*P* = .0055), indicating distinct performance variations. Similarly, HIPT4K showed a significant difference from CTransPath (*P* = .0034) but had weaker evidence against SimCLR (*P* = .1330) and RetCCL (*P* = .0617), suggesting potential similarity. The highest *P*-values were observed between RetCCL and Imagenet (*P* = .3374) and SimCLR versus RetCCL (*P* = .3128), implying no statistically significant performance differences between these models.

Beyond the quantitative performance evaluation of our approach, we further assessed attention heatmaps with two neuropathologists (W.R.B. and H.H.) to enhance the AI model interpretability and understand any underlying decision-driving morphological features. Such analysis holds promises to assist AI-guided clinical diagnoses and contribute to a broader comprehension of morpho-molecular correlations. Neuropathological assessment of our attention heatmap results (Figures 3a and d) for the correctly identified cases revealed that the areas of high attention were associated with regions of morphologic features well-known to belong to these tumors. Specifically, correctly predicted IDHwt cases exhibit pleomorphism, infiltrating cells, microvascular proliferation, mitotic activity in vessels, hyperchromatic nuclei, and the presence of “ghost” cells with necrotic debris and cellular heterogeneity. Correctly predicted IDHmut cases include gemistocytic cells, microcystic changes, oligodendrocyte-like cells, mixed cytoplasmic clearing, and myxoid appearance. These features align with the histological profiles of IDHmut gliomas, indicating that both the algorithm and neuropathologists could recognize the subtle yet distinct morphological traits associated with IDH. Attention heatmaps for IDHwt cases incorrectly predicted as IDHmut identify artifacts, infiltrating cortex, nonspecific inflammatory cells, fibrillary processes, and “moth-eaten” tissue, with open chromatin in tumor cells, suggesting that the algorithm might have been misled by ambiguous or atypical features (Figure 3b). In contrast, cases misclassified as IDHwt, present gemistocytic changes, dense cells with heterogeneous hyperchromatism, and features suggestive of prior radiation (Figure 3c). Given the intensive nature of the review process and the inherent subjectivity of this approach, we further present an automated deep learning model to quantify cells within high-attention regions (details in the Supplementary Material [Section 3]).

We want to acknowledge certain limitations of our study. First, our computational models are based on retrospective datasets and developed while considering IDH alterations as a group of IDHmut rather than a way to individually assess IDH1, IDH2, or specific subtypes of IDH1 mutations. It is imperative to recognize the need for comprehensive validation on larger, multicentric datasets to ensure our approach’s broad applicability and robustness. Ideally, an equal distribution of cases with varying IDH mutation along with grade and histology would result in the optimal AI model training configuration. Even though our results on the complete dataset cohort achieved a consistent AUC of 0.95 and above, it is worth noting that the performance significantly drops when we analyze our model’s performance on “molecular” GBM cases, which represented a much smaller sample size. Additionally, we acknowledge the limitation that the current work focused on adult diffuse gliomas (ie, astrocytoma, IDH-mutant; oligodendroglioma, IDH-mutant, 1p/19q-codeleted; glioblastoma, IDH-wildtype—as defined by Louis et al.^3^) does not address the clinical applicability of the model in distinguishing other typical IDHwt tumors with similar histological features, such as pilocytic astrocytoma. To address this, future studies could focus specifically on this classification endpoint, incorporating a larger and more balanced dataset to improve model generalizability.

Our AI approach classifies adult diffuse gliomas as either IDHmut or IDHwt solely from H&E-stained slides. This study presents a pioneering approach for applying AI algorithms in glioma research by employing a weakly supervised computational approach to classify molecular biomarkers. Our findings underscore the efficacy of DL models solely based on WSI, with the potential of achieving optimal performance in exceptional cases like “molecular” GBMs, where gold-standard microscopic evaluation remains inconclusive. We envision implementing these AI pipelines seamlessly to assist pathologists in clinical practice. AI diagnostic assistance can revolutionize the accuracy of diagnoses, enhance prognostic stratification, and ultimately elevate the quality of decision-making, all aiming to improve patient outcomes. This study opens doors to a future where AI-driven insights will advance our understanding and management of gliomas.

## Supplementary Material

vdaf140_suppl_Supplementary_Materials

## Data Availability

TCGA dataset is available through the TCIA portal. UPHS dataset is a private dataset. EBRAINS is publicly available through The Digital Brain Tumor Atlas. The code for this study is available through https://github.com/IUCompPath/IDHclassifier.

## References

[1] Ostrom QT , Gittleman H, Truitt G, et al CBTRUS statistical report: primary brain and other central nervous system tumors diagnosed in the United States in 2011–2015. Neuro-Oncology. 2018;20(suppl 4):iv1iv1–iviv86. 30445539 10.1093/neuonc/noy131 PMC6129949

[2] Louis DN , Ohgaki H, Wiestler OD, et al The 2007 WHO classification of tumours of the central nervous system. Acta Neuropathol. 2007;114(2):97–109. 17618441 10.1007/s00401-007-0243-4 PMC1929165

[3] Louis DN , Perry A, Wesseling P, et al The 2021 WHO classification of tumors of the central nervous system: a summary. Neuro-Oncology. 2021;23(8):1231–1251. 34185076 10.1093/neuonc/noab106 PMC8328013

[4] Jakola AS , Skjulsvik AJ, Myrmel K, et al Surgical resection versus watchful waiting in low-grade gliomas. Ann Oncol. 2017;28(8):1942–1948. 28475680 10.1093/annonc/mdx230 PMC5834105

[5] Wijnenga MM , French PJ, Dubbink HJ, et al The impact of surgery in molecularly defined low-grade glioma: an integrated clinical, radiological, and molecular analysis. Neuro-Oncology. 2018;20(1):103–112. 29016833 10.1093/neuonc/nox176 PMC5761503

[6] Gao Y , Weenink B, van den Bent M, et al Expression-based intrinsic glioma subtypes are prognostic in low-grade gliomas of the EORTC 22033-26033 clinical trial. Eur J Cancer. 2018;94:168–178. 29571083 10.1016/j.ejca.2018.02.023

[7] Bunse L , Pusch S, Bunse T, et al Suppression of antitumor T cell immunity by the oncometabolite (R)-2-hydroxyglutarate. Nat Med. 2018;24(8):1192–1203. 29988124 10.1038/s41591-018-0095-6

[8] Draaisma K , Chatzipli A, Taphoorn M, et al Molecular evolution of IDH wild-type glioblastomas treated with standard of care affects survival and design of precision medicine trials: a report from the EORTC 1542 study. J Clin Oncol. 2020;38(1):81–99. 31743054 10.1200/JCO.19.00367

[9] Pirozzi CJ , Yan H. The implications of IDH mutations for cancer development and therapy. Nat Rev Clin Oncol. 2021;18(10):645–661. 34131315 10.1038/s41571-021-00521-0

[10] Olar A , Wani KM, Alfaro-Munoz KD, et al IDH mutation status and role of WHO grade and mitotic index in overall survival in grade II–III diffuse gliomas. Acta Neuropathol. 2015;129(4):585–596. 25701198 10.1007/s00401-015-1398-z PMC4369189

[11] Perry A , Wesseling P. Histologic classification of gliomas. Handbook Clin Neurol. 2016;134:71–95. 10.1016/B978-0-12-802997-8.00005-0 26948349

[12] Weller M , Wick W, Aldape K, et al Glioma. Nat Rev Dis Primers. 2015;10(33):1–18. 10.1038/nrdp.2015.17 27188790

[13] Liechty B , Xu Z, Zhang Z, et al Machine learning can aid in prediction of IDH mutation from H&E-stained histology slides in infiltrating gliomas. Sci Rep. 2022;12(1):22623. 36587030 10.1038/s41598-022-26170-6 PMC9805452

[14] Guo X , Gu L, Li Y, et al Histological and molecular glioblastoma, IDH-wildtype: a real-world landscape using the 2021 WHO classification of central nervous system tumors. Front Oncol. 2023;13(1200815):1–14. 10.3389/fonc.2023.1200815 PMC10358772 37483487

[15] Agarwal A , Edgar MA, Desai A, et al Molecular GBM versus histopathological GBM: radiology-pathology-genetic correlation and the new WHO 2021 definition of glioblastoma. AJNR Am J Neuroradiol. 2024;45(8):1006–1012. 38438167 10.3174/ajnr.A8225 PMC11383408

[16] Choi YS , Bae S, Chang JH, et al Fully automated hybrid approach to predict the IDH mutation status of gliomas via deep learning and radiomics. Neuro-Oncology. 2020;23(2):304–313. 10.1093/neuonc/noaa177 PMC7906063 32706862

[17] Chakrabarty S , LaMontagne P, Shimony J, Marcus DS, Sotiras A. MRI-based classification of IDH mutation and 1p/19q codeletion status of gliomas using a 2.5D hybrid multi-task convolutional neural network. Neurooncol. Adv.. 2023;5(1):vdad023. 37152810 10.1093/noajnl/vdad023 PMC10162113

[18] Bakas S , Vollmuth P, Galldiks N, et al; Response Assessment in Neuro Oncology (RANO) group. Artificial Intelligence for Response Assessment in Neuro Oncology (AI-RANO), part 2: recommendations for standardisation, validation, and good clinical practice. Lancet Oncol. 2024;25(11):e589–e601. 39481415 10.1016/S1470-2045(24)00315-2 PMC12007431

[19] Villanueva-Meyer JE , Bakas S, Tiwari P, et al; Response Assessment in Neuro Oncology (RANO) group. Artificial Intelligence for Response Assessment in Neuro Oncology (AI-RANO), part 1: review of current advancements. Lancet Oncol. 2024;25(11):e581–e588. 39481414 10.1016/S1470-2045(24)00316-4 PMC12045294

[20] Sanai N , Berger MS. Surgical oncology for gliomas: the state of the art. Nat Rev Clin Oncol. 2018;15(2):112–125. 29158591 10.1038/nrclinonc.2017.171

[21] Capper D , Zentgraf H, Balss J, Hartmann C, von Deimling A. Monoclonal antibody specific for IDH1 R132H mutation. Acta Neuropathol. 2009;118(5):599–601. 19798509 10.1007/s00401-009-0595-z

[22] Louis DN , Perry A, Reifenberger G, et al The 2016 World Health Organization classification of tumors of the central nervous system: a summary. Acta Neuropathol. 2016;131(6):803–820. 27157931 10.1007/s00401-016-1545-1

[23] Han S , Liu Y, Cai SJ, et al IDH mutation in glioma: molecular mechanisms and potential therapeutic targets. Br J Cancer. 2020;122(11):1580–1589. 32291392 10.1038/s41416-020-0814-x PMC7250901

[24] Melhem JM , Detsky J, Lim-Fat MJ, Perry JR. Updates in IDH-wildtype glioblastoma. Neurotherapeutics. 2022;19(6):1705–1723. 35641844 10.1007/s13311-022-01251-6 PMC9154038

[25] Baheti B , Innani S, Mehdiratta G, Nasrallah MP, Bakas S. P13.13.b Interpretable whole slide image prognostic stratification of glioblastoma patients furthering disease understanding. Neuro-Oncology. 2023;25(Suppl 2):ii103–ii104.

[26] Baheti B , Innani S, Nasrallah MP, Bakas S. OS03.6.a Unsupervised clustering of morphology patterns on whole slide images guide prognostic stratification of glioblastoma patients. Neuro-Oncology. 2023;25(Suppl 2):ii15–ii15. 10.3389/fnins.2024.1304191 PMC11146603 38831756

[27] Baheti B , Innani S, Nasrallah M, Bakas S. Prognostic stratification of glioblastoma patients by unsupervised clustering of morphology patterns on whole slide images furthering our disease understanding. Front Neurosci. 2024;18(1304191):1–12. 10.3389/fnins.2024.1304191 PMC11146603 38831756

[28] Baheti B , Rai S, Innani S, et al Multimodal explainable artificial intelligence for prognostic stratification of patients with glioblastoma. Modern Patholo. 2025;38(9):100797. https://doi.org/10.1016/j.modpat.2025.100797. PMC12212623 40419087

[29] Innani S , Baheti B, Nasrallah MP, et al Path-39. Interpretable IDH classification from H&E-stained histology slides. Neuro-Oncology. 2023;25(Suppl 5):v177–v177.

[30] Innani S , Baheti B, Nasrallah MP, et al Weakly supervised IDH-status glioma classification from H&E-stained whole slide images. In: 2024 IEEE International Symposium on Biomedical Imaging (ISBI); 2024; pp. 1–5.

[31] Innani S , Nasrallah MP, Bell WR, et al Multi-scale whole slide image assessment improves deep learning based WHO 2021 glioma classification. In: Proceedings of the MICCAI Workshop on Computational Pathology; 2024 Oct 6; vol. 254. PMLR; pp. 142–153.

[32] Lu MY , Williamson DF, Chen TY, et al Data-efficient and weakly supervised computational pathology on whole-slide images. Nat Biomed Eng. 2021;5(6):555–570. 33649564 10.1038/s41551-020-00682-w PMC8711640

[33] Saldanha O , Löffler C, Niehues J, et al Self-supervised attention-based deep learning for pan-cancer mutation prediction from histopathology. npj Precis Oncol. 2023;7(1):35:16. 36977919 10.1038/s41698-023-00365-0 PMC10050159

[34] Chen RJ , Ding T, Lu MY, et al Towards a general-purpose foundation model for computational pathology. Nat Med. 2024;30(3):850–862. 38504018 10.1038/s41591-024-02857-3 PMC11403354

[35] Wölflein G , Ferber D, Meneghetti AR, et al A good feature extractor is all you need for weakly supervised pathology slide classification. Computer Vision-ECCV 2024 Workshops 2025;15638:68–87.

[36] Lazard T , Bataillon G, Naylor P, et al Deep learning identifies morphological patterns of homologous recombination deficiency in luminal breast cancers from whole slide images. Cell Rep Med. 2022;3(12):100872. 36516847 10.1016/j.xcrm.2022.100872 PMC9798078

[37] Wang X , Du Y, Yang S, et al RETCCL: Clustering-guided contrastive learning for whole-slide image retrieval. Med Image Anal. 2023;83:102645. 36270093 10.1016/j.media.2022.102645

[38] Kang M , Song H, Park S, et al Benchmarking self-supervised learning on diverse pathology datasets. In: 2023 IEEE/CVF Conference on Computer Vision and Pattern Recognition (CVPR); 2023; Los Alamitos, CA, USA: IEEE Computer Society; pp. 3344–3354.

[39] Wang X , Yang S, Zhang J, et al Transformer-based unsupervised contrastive learning for histopathological image classification. Med Image Anal. 2022;81:102559. 35952419 10.1016/j.media.2022.102559

[40] Chen RJ , Chen C, Li Y, et al Scaling vision transformers to gigapixel images via hierarchical self-supervised learning. In: 2022 IEEE/CVF Conference on Computer Vision and Pattern Recognition (CVPR); 2022; Los Alamitos, CA, USA: IEEE Computer Society; pp. 16123–16134.

[41] Ciga O , Xu T, Martel AL, et al Self-supervised contrastive learning for digital histopathology. Mach Learn Appl. 2022;7:100198.

[42] Deng J , Dong W, Socher R, et al ImageNet: A large-scale hierarchical image database. In: 2009 IEEE Conference on Computer Vision and Pattern Recognition (CVPR); 2009; pp. 248–255.

[43] Baheti B , Rai S, Innani S, et al EPCO-15. Detecting histologic & clinical glioblastoma patterns of prognostic relevance. Neuro-Oncology. 2023;25(Suppl 5):v126–v126.

[44] Clark K , Vendt B, Smith K, et al The cancer imaging archive (TCIA): Maintaining and operating a public information repository. J Digit Imaging. 2013;26(6):1045–1057. 23884657 10.1007/s10278-013-9622-7 PMC3824915

[45] Roetzer-Pejrimovsky T , Moser AC, Atli B, et al The digital brain tumour atlas, an open histopathology resource. Sci Data. 2022;9(1):55. 35169150 10.1038/s41597-022-01157-0 PMC8847577

[46] Dosovitskiy A , Beyer L, Kolesnikov A, et al An image is worth 16x16 words: transformers for image recognition at scale. International Conference on Learning Representations. 2021.

[47] Chang K , et al; TCGA Research Network, Genome Characterization Center. The Cancer Genome Atlas Pan-Cancer analysis project. Nat Genet. 2013;45(10):1113–1120. 24071849 10.1038/ng.2764 PMC3919969

[48] Kim YJ , Jang H, Lee K, et al PAIP 2019: liver cancer segmentation challenge. Med Image Anal. 2021;67:101854. 33091742 10.1016/j.media.2020.101854

[49] Chen X , Xie S, He K, et al An empirical study of training self-supervised vision transformers. In: 2021 IEEE/CVF International Conference on Computer Vision (ICCV). Los Alamitos, CA, USA: IEEE Computer Society; 2021. p. 9620–9629.

[50] Liu Z , Lin Y, Cao Y, et al Swin transformer: hierarchical vision transformer using shifted windows. In: 2021 IEEE/CVF International Conference on Computer Vision (ICCV). 2021. p. 9992–10002.

[51] He K , Zhang X, Ren S, et al Deep residual learning for image recognition. In: 2016 IEEE Conference on Computer Vision and Pattern Recognition (CVPR); 2016; pp. 770–778.

[52] Caron M , Touvron H, Misra I, et al Emerging properties in self-supervised vision transformers. In: 2021 IEEE/CVF International Conference on Computer Vision (ICCV). 2021. p. 9630–9640.

[53] Ilse M , Tomczak J, Welling M, et al Attention-based deep multiple instance learning. In: International Conference on Machine Learning. PMLR; 2018. p. 2127–2136.

[54] McHugh ML. Interrater reliability: the kappa statistic. Biochemia Medica. 2012;22(3):276–282. 23092060 PMC3900052

[55] Momeni A , Thibault M, Gevaert O. Deep recurrent attention models for histopathological image analysis. bioRxiv. 2018.

[56] Jiang S , Zanazzi GJ, Hassanpour S. Predicting prognosis and IDH mutation status for patients with lower-grade gliomas using whole slide images. Sci Rep. 2021;11(1):16849. 34413349 10.1038/s41598-021-95948-x PMC8377095

[57] Liu S , Shah Z, Sav A, et al Isocitrate dehydrogenase (IDH) status prediction in histopathology images of gliomas using deep learning. Sci Rep. 2020;10(1):7733. 32382048 10.1038/s41598-020-64588-y PMC7206037

[58] Hewitt KJ , Löffler CML, Muti HS, et al Direct image to subtype prediction for brain tumors using deep learning. Neurooncol. Adv. 2023;5(1):vdad139. 38106649 10.1093/noajnl/vdad139 PMC10724115

