# Supplementary material for: Interpretable artificial intelligence based determination of glioma IDH mutation status directly from histology slides
Source: Neurooncol Adv. 2025 Jul 11;7(1):vdaf140. doi: 10.1093/noajnl/vdaf140 (PMC12290450; doi:10.1093/noajnl/vdaf140)
Supplement: vdaf140_suppl_Supplementary_Materials [file vdaf140_suppl_supplementary_materials.docx]

Interpretable artificial intelligence-based determination of glioma IDH mutation status directly from histology slides

***Supplementary Material***

1. **Weakly Supervised Learning**

After feature extraction, We further employ a MIL with an attention mechanism [[1](#_bookmark40), [2](#_bookmark61)](AttMIL), to consolidate the patch-level features into comprehensive slide-level representations. Following the MIL assumption [[3](#_bookmark62)], where each patch within a WSI is treated as an instance and the WSI itself as a bag, our model processes WSIs represented as feature vectors of size *N ×* 1024. Initially, each 1024-dimensional feature vector undergoes dimensionality reduction to size of 512, through a trainable fully connected (FC) layer. This reduced feature vector is then forwarded to the attention network, which consists of two parallel FC layers employing Tanh and Sigmoid activations respectively. The outputs of these layers each of size *N ×* 256, are element-wise multiplied to derive attention scores for each patch and passing through another linear layer resulting in an output of size *N ×* 1. The MIL model employs an attention-based pooling mechanism that assigns an attention weight $\alpha_{i}$ to each patch $x_{i}$, quantifying its importance in determining the final classification outcome. The slide-level representation is computed as

$$z =\sum_{i=1}^{N} \alpha_{i}x_{i}$$

where *N* is the number of patches in the slide. A separate FC layer with softmax activation is used to predict a class probability for each patch based on the 512-dimensional features, resulting in an output of size *N ×* 3. The attention scores *N ×* 1 are used as weights to aggregate the patch-level predictions into a single prediction for the entire WSI, ensuring that more important patches have a greater influence on the final classification. Additionally, a binary clustering layer with 512 hidden neurons combined with an SVM loss function, follows the initial fully connected layer to refine specific features [[1](#_bookmark40)]. This layer capitalizes on features from patches with high attention scores to construct a rich slide-level feature space that effectively distinguishes between positive and negative instances for the two classes. Subsequently, a dense layer aggregates predictions from patches based on their attention scores, effectively filtering out contribution from less significant patches, which are pivotal for classification of each WSI into one of two categories: IDHwt or IDHmut. In the feature aggregation phase, to evaluate the impact of different MIL strategies, four different : MeanMIL[[4](#_bookmark63)], & MaxMIL[[4](#_bookmark63)] employing mean-pool and max-pooling respectively, DSMIL[[5](#_bookmark64)] combining dual-stream max-pooling with attention scoring, TransMIL[[6](#_bookmark65)] leveraging transformer based self-attention model. This comprehensive evaluation allows us to assess the effectiveness of different MIL strategies in capturing relevant histologic patterns for IDH classification, providing a robust and interpretable deep learning approach for direct histology-based prediction. For training, the model was trained using the Adam optimizer with a learning rate of 0.0001, and a dropout rate of 0.25 to prevent overfitting. Early stopping was applied to halt training once the validation loss no longer improved after 20 consecutive epochs. The model was trained for a maximum of 200 epochs, with model checkpoints saved based on the best validation performance. Weighted cross-entropy loss is implemented to mitigate the class imbalance problem. Performance was evaluated using two primary metrics: the Area Under the Curve (AUC) and the Youden Index. The AUC is a standard measure of classification performance, representing the model’s ability to discriminate between the two classes (IDHwt vs. IDHmut). The Youden Index is defined as *sensitivity* + *Specificity −* 1 and it provides a balanced measure of the model’s performance across both classes by considering both false positives and false negatives. These metrics were calculated for each fold during the cross-validation process and averaged to give a final evaluation score.

1. **Ablation results of MILs**

Table 1: Performance across subcohorts with different MILs. Values depict mean ± standard deviation.

1. Average AUC values with different MILs

| Model | 10-fold Cross Validation | | |
| --- | --- | --- | --- |
|  | Sub cohort #1 | Sub cohort #2 | Sub cohort #3 |
| MaxMIL | 0.9545 ± 0.0205 | 0.9451 ± 0.0357 | 0.9350 ± 0.0549 |
| MeanMIL | 0.9682 ± 0.0089 | 0.9571 ± 0.0295 | 0.9633 ± 0.0269 |
| TransMIL | 0.9611 ± 0.0249 | 0.9355 ± 0.0458 | 0.9508 ± 0.0552 |
| DSMIL | 0.9623 ± 0.0149 | 0.9491 ± 0.0291 | 0.9549 ± 0.0555 |
| AttMIL | **0.9716 ± 0.0113** | **0.9616 ± 0.0342** | **0.9689 ± 0.0211** |

1. Average Youden Index values with different MILs

| Model | 10-fold Cross Validation | | |
| --- | --- | --- | --- |
|  | Sub cohort #1 | Sub cohort #2 | Sub cohort #3 |
| MaxMIL | 0.7881 ± 0.0573 | 0.7581 ± 0.0642 | 0.6639 ± 0.2315 |
| MeanMIL | 0.8150 ± 0.0386 | 0.7771 ± 0.1039 | 0.6751 ± 0.2110 |
| TransMIL | 0.7641 ± 0.0947 | 0.6966 ± 0.1288 | 0.7170 ± 0.1947 |
| DSMIL | 0.8129 ± 0.0385 | 0.7656 ± 0.0943 | 0.7082 ± 0.2419 |
| AttMIL | **0.8189 ± 0.0300** | **0.8093 ± 0.0735** | **0.7712 ± 0.1669** |

We have conducted experiments with several recent MIL-based approaches, including DSMIL, MaxMIL, MeanMIL, TransMIL, and AttMIL. These results demonstrate the performance of these methods using the best-performing feature extractor, UNI. As in Table 1 results, AttMIL consistently outperformed the other MIL approaches. Additionally, the comparison between MIL methods and Foundation Models (FMs) indicates that FMs are more influential in driving performance gains than the choice of aggregation method. While MIL methods show promising results, FMs consistently outperform them, indicating that feature extraction is more important than aggregation strategies for model performance.

Among the various MIL approaches, AttMIL consistently showed the highest performance across all three cohorts, achieving AUCs of 0.9716, 0.9616, and 0.9689 for Cohort1, Cohort2, and Cohort3, respectively. The DSMIL approach followed closely, with AUCs of 0.9623, 0.9491, and 0.9549 across the three cohorts. The MaxMIL method demonstrated slightly lower performance, with AUCs of 0.9545, 0.9451, and 0.9350 for the respective cohorts. MeanMIL achieved AUC values of 0.9682, 0.9571, and 0.9633, demonstrating competitive results. Lastly, Trans-MIL exhibited the lowest performance, with AUCs of 0.9611, 0.9355, and 0.9508. These results highlight the superior performance of the AttMIL method in accurately distinguishing IDHmut from IDHwt gliomas across all three TCGA cohorts.

For the ablation study, we also evaluated the contribution of the attention mechanism. In these experiments, we removed the attention mechanism from the Att-MIL model and compared its performance to the full AttMIL model. The ablation results, as in Table 1, show that the attention mechanism significantly improves performance and interpretability, confirming its essential role in the model. AttMIL stands out for its ability to generate interpretable attention maps, which is not possible with basic pooling methods. The attention scores generated by AttMIL can be visualized into heatmaps, which highlight the most relevant patches in the WSI, enhancing model interpretability.

While MeanMIL and MaxMIL rely on basic pooling operations such as mean and max pooling, TransMIL utilizes a transformer-based self-attention mechanism, and DSMIL employs dual-stream max-pooling with attention scoring. Despite implementing different MIL methodologies, the performance differences compared to foundation models (FMs) are minimal, suggesting that FMs offer a notable advancement over traditional MIL approaches. Additionally, while MIL methods show promising results, AttMIL consistently outperforms the other MIL strategies, highlighting its superior efficacy. Although TransMIL and DSMIL are theoretically promising, they did not yield the anticipated improvements.

1. **Hovernet Interpretations**

Along with the WSI-level interpretation, we also assessed the distribution of different cell types present in the high attention regions. A pre-trained cell detection and segmentation model, HoverNet[7], is utilized for this cell quantification on the top 50 tiles with the highest attention scores to detect nuclear cells and distinguish between five distinct cell types: tumor, lymphocytes, stromal cells, epithelial, and necrotic cells[10]. Furthermore, we also conducted a p-value-based statistical analysis using an independent t-test between cell counts among three different classes: IDHwt, IDHmut, and Molecular GBM to discern and quantify variations in cellular composition across these classes and provide valuable insights into the distinctive cellular profiles. Figure 1a offers visual insights into the detection of various cell types present in high-attention regions. Quantification of these cell types provides detailed cell-level quantification for IDHwt, IDHmut, and Molecular GBM (histologically lower-grade glioma with IDH mutation status as IDHwt, without histological evidence of Grade 4 Glioblastoma) as shown in Figure 1b, enhancing the understanding of morphological characteristics and potential clinical implications.


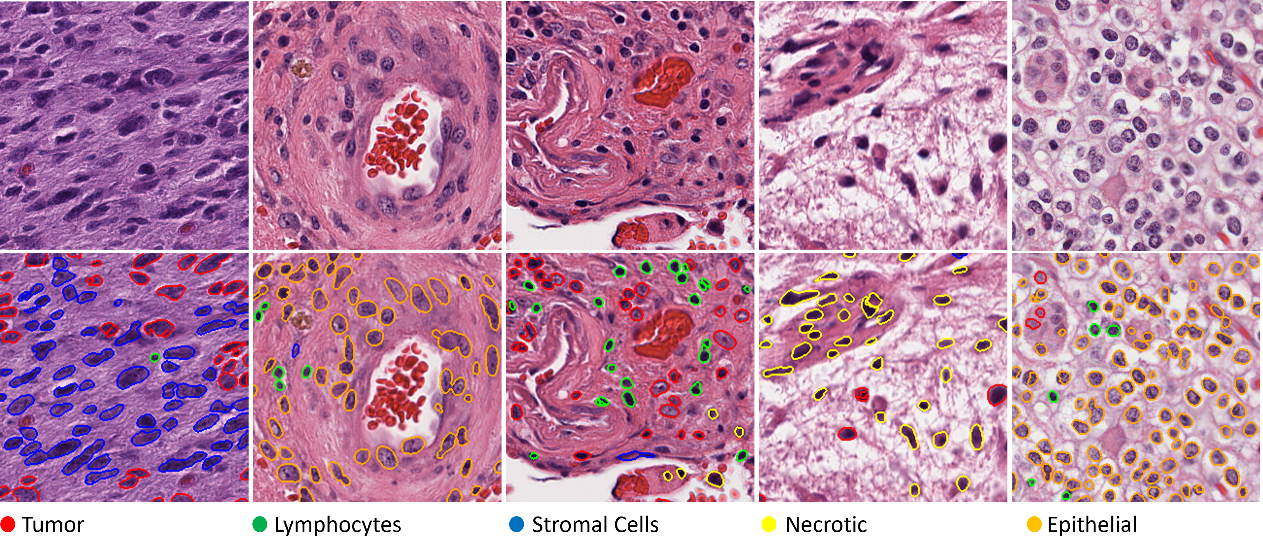


(a) Cell detection and classification with pre-trained HoverNet to identify five different kinds of cells i.e. tumor, lymphocytes, stromal, necrotic, and epithelial.


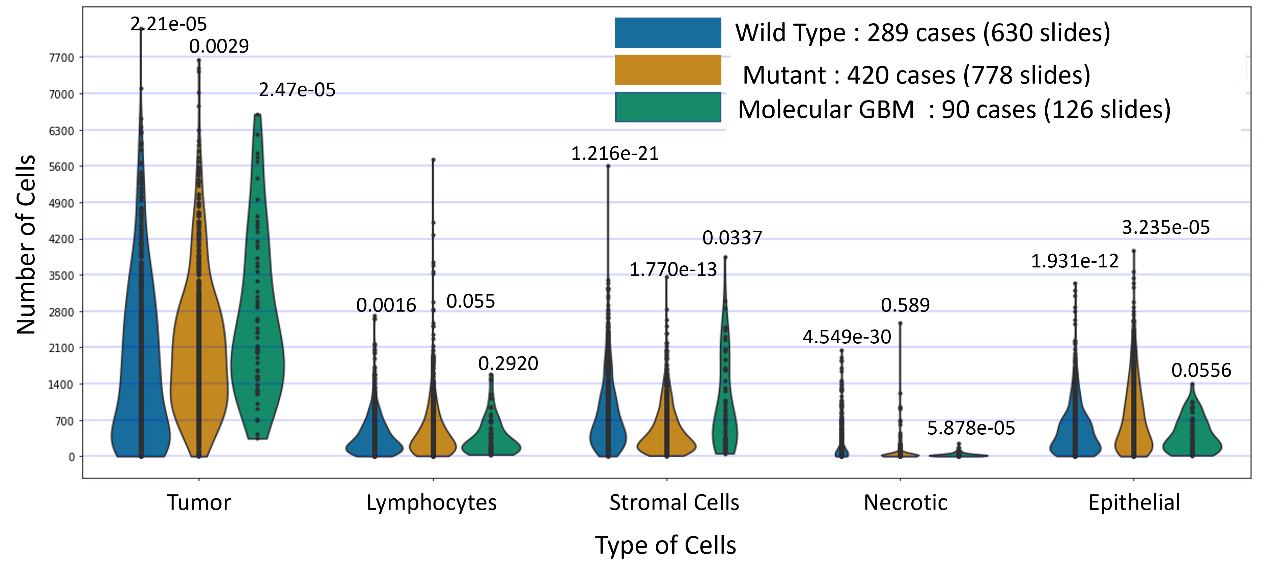


(b) Quantitative distribution of different cell types in IDHwt, IDHmut, and Molecular GBM for complete dataset cohort. A statistical test conducted using independent t-test and p-value is indicated between three pais (IDHwt-IDHmut; IDHmut-molecular GBM; and molecular GBM-IDHwt) above each violin plot.

Fig. 1: Cell classification and quantification

Global morphological patterns were analyzed by quantifying cells in regions with high attention from IDHwt glioblastoma with or without lower-grade histologic features and IDHmut gliomas, as shown in Figure 1b. This comprehensive analysis of cellular composition at the tile level further contributed to our understanding of the underlying tissue characteristics. The comparison of violin plots for IDHwt with IDHmut tumors (excluding GBM with lower-grade histology) shows that the former exhibited a lower distribution of tumor cells (p = 2.21e-05) and necrotic cells (p = 1.93e-12) and a higher distribution of epithelial (p = 4.549e-30) and stromal cell-type tissue (p = 1.21e-21), while showing a similar distribution for inflammatory cells (p = 0.002). However, our neuropathologist’s analysis of violin plots suggests a potential lack of clinical relevance. While looking closely at the cell segmentation results with neuropathologists, we realized that this could be due to inaccurate cell classification in some cases. This highlights the limited generalizability of the HoverNet model to brain glioma, as it was trained on 19 different organs. Accurate cell segmentation and classification algorithms would lead to more accurate downstream analyses and improved clinical significance. Hovernet model with pre-trained weights are available at <https://github.com/vqdang/hover_net>.

1. **Statistical Significance**

Table [2](#_bookmark1) summarizes the statistical significance across all the considered feature extractors, following the DELPHI-based recommendations for image analysis validation [8,9], incorporating i) algorithmic ranking, and ii) statistical significance testing. For this analysis we divided the test data into multiple non-overlapping subsets, ensuring balanced class representation. We then computed an average rank for each of the subsets across all multi-scale approaches and aggregated these average rankings to produce a conclusive overall ranking. All approaches were then placed in a ranked order and their average rankings were randomly permuted (i.e., 100,000 permutations), in a pair-wise manner. Corresponding pairwise p-values shown in Table [2](#_bookmark1) were computed to determine the pair-wise statistical significance and report actual differences between the ordered ranked approaches. These p-values are reported in an upper triangular matrix (Table [2](#_bookmark1)) revealing the statistical difference between model performances. Lower p-values (≤ 0.05) suggest significant differences, while higher values indicate similarity. UNI exhibits significant differences compared to LunitViT (p=0.0270) and CTransPath (p=0.0055), suggesting that these models perform distinctly. Similarly, HIPT4K shows a significant difference from CTransPath (p=0.0034) but has weaker evidence against SimCLR (p=0.1330) and RetCCL (p=0.0617), indicating potential similarity in performance. The highest p-values are observed between RetCCL and Imagenet (p=0.3374) and SimCLR vs. RetCCL (p=0.3128), suggesting that these models do not exhibit statistically significant performance differences.

Table 2: Pairwise statistical comparison based on permutation testing across different FMs.

|  | UNI | LunitViT | CTranspath | HIPT4K | SimCLR | RetCCL | Imagenet |
| --- | --- | --- | --- | --- | --- | --- | --- |
| UNI |  | 0.0270 | 0.0055 | 0.0000 | 0.0000 | 0.0000 | 0.0000 |
| LunitViT |  | 0.3037 | | 0.0009 | 0.0000 | 0.0000 | 0.0000 |
| CTransPath |  |  | | 0.0034 | 0.0001 | 0.0000 | 0.0000 |
| HIPT4k |  |  | |  | 0.1330 | 0.0617 | 0.0254 |
| SimCLR |  |  |  |  |  | 0.3128 | 0.1859 |
| RetCCL |  |  |  |  |  |  | 0.3374 |
| Imagenet |  |  | |  | | |  |

1. **Keywords from Heatmap assessments**

**For inter-rater reliability assessment, we employed Cohen’s kappa coefficient using a predefined set of histopathological keywords that represent key morphological and architectural features relevant to glioma diagnosis. The selected terms included: tumor cells, pleomorphic cells, gemistocytic cells, oligodendrocyte, astrocytic, neoplastic, hyperchromatic nuclei, eosinophilic cytoplasm, cytoplasmic clearing, fibrillary, monotonous cells, microcystic change, palisading necrosis, microvascular proliferation, infiltrating cortex/white matter, mixoid material, perinuclear halos (“hellos”), vascular channels/vessels/ropy vessels, lumens, epithelial cells, and cortillary artifact. These features were chosen based on their relevance to the histologic characterization of gliomas and were consistently applied across annotators to assess agreement.**

1. **Supplementary Table**

Table 3: Comparison of Approaches and Results from different literature

| Study | Approach | Dataset | Results |
| --- | --- | --- | --- |
| Momeni et al. | Deep Recurrent   Attention Models | TCGA-GBM, TCGA-LGG:  974 WSIs | Accuracy_test_=79% |
| Liu et al. | GAN approach   for data | TCGA-GBM, TCGA-LGG:  266 WSIs | AUC_test_=92.7% |
| Jiang et al. | Training on Grade II,  Test on Grade III | TCGA-LGG:  524 WSIs | AUC_test_=81.4% |
| Liechty et al. | Multiscale ensemble | TCGA-GBM, TCGA-LGG:  801 WSIs | Accuracy_test_=94.9% |
| Hewitt et al. | MIL | Training - UCL:  1882 WSI  Holdout - TCGA-GBM, TCGA-LGG: 864 WSI | AUC_UCL_=95%  AUC_TCGA_=90% |
| Ours | **AttMIL** | **Training - TCGA-GBM, TCGA-LGG: 1534 WSIs**  **External Cohort 1:**  **EBRAINS 794 WSI**  **External Cohort 2:**  **UPHS 114 WSI** | **AUC_TCGA_=97.16%**  **AUC_UPHS_=97.37%**  **AUC_EBRAINS_=95.34%** |

Table 4: Comparison of model performance across grade

| ***Tumor Grade*** | ***IDH Status*** | ***Classification*** | ***Model Accuracy*** |
| --- | --- | --- | --- |
| *Grade 2* | *IDH-mut* | *Traditional LGG* | *94%* |
| *Grade 3* | *IDH-mut* | *Traditional LGG* | *92%* |
| *Grade 4* | *IDH-mut* | *Historical GBM* | *62%* |
| *Grade 4* | *IDH-wt* | *Traditional GBM* | *94%* |
| *Grade 3* | *IDH-wt* | *Molecular GBM* | *57%* |
| *Grade 2* | *IDH-wt* | *Molecular GBM* | *56%* |

Reference:

1. Lu MY, Williamson DF, Chen TY, Chen RJ, Barbieri M, Mahmood F. Data-efficient and weakly supervised computational pathology on whole-slide images. Nat Biomed Eng. 2021;5(6):555-570.
2. Saldanha O, Löffler C, Niehues J, Treeck M, Seraphin T, Hewitt K, Cifci D, Veldhuizen G, Ramesh S, Pearson A, Kather J. Self-supervised attention-based deep learning for pan-cancer mutation prediction from histopathology. NPJ Precis Oncol. 2023;7:16.
3. O. Maron and T. Lozano-P´erez, “A framework for multiple-instance learning,” Advances in neural informa- tion processing systems, vol. 10, 1997.
4. Y. Wang, J. Li, and F. Metze, “A comparison of five multiple instance learning pooling functions for sound event detection with weak labeling,” in ICASSP 2019-2019 IEEE International Conference on Acoustics, Speech and Signal Processing (ICASSP). IEEE, 2019, pp. 31–35.
5. B. Li, Y. Li, and K. W. Eliceiri, “Dual-stream multiple instance learning network for whole slide image classi- fication with self-supervised contrastive learning,” in Proceedings of the IEEE/CVF conference on computer vision and pattern recognition, 2021, pp. 14 318–14 328.
6. Z. Shao, H. Bian, Y. Chen, Y. Wang, J. Zhang, X. Ji et al., “Transmil: Transformer based correlated multiple instance learning for whole slide image classification,” Advances in neural information processing systems, vol. 34, pp. 2136–2147, 2021.
7. Graham S, Vu QD, Raza SEA, Azam A, Tsang YW, Kwak JT, Rajpoot N. Hover-net: Simultaneous segmentation and classification of nuclei in multi-tissue histology images. Med Image Anal. 2019; 58:101563.
8. Maier-Hein, L., Reinke, A., Godau, P., Tizabi, M.D., Buettner, F., Christodoulou, E., Glocker, B., Isensee, F., Kleesiek, J., Kozubek, M., et al.: Metrics reloaded: recommendations for image analysis validation. Nature methods 21(2), 195–212 (2024)
9. Reinke, A., Tizabi, M.D., Baumgartner, M., Eisenmann, M., Heckmann-N¨otzel, D., Kavur, A.E., R¨adsch, T., Sudre, C.H., Acion, L., Antonelli, M., et al.: Understanding metric-related pitfalls in image analysis validation. Nature methods 21(2), 182–194 (2024).
10. Chen, R. J., Lu, M. Y., Williamson, D. F., Chen, T. Y., Lipkova, J., Noor, Z., ... & Mahmood, F. (2022). Pan-cancer integrative histology-genomic analysis via multimodal deep learning. *Cancer cell*, *40*(8), 865-878.

**Supplementary Figure Legends**

Supplementary Fig. 1: Cell classification and quantification

**Supplementary Table Legends**

Supplementary Table 1: Performance across subcohorts with different MILs. Values depict mean ± standard deviation.

Supplementary Table 2: Pairwise statistical comparison based on permutation testing across different FMs.

Supplementary Table 3: Comparison of Approaches and Results from different literature.

Supplementary Table 4: Comparison of model performance across grade
